# Olig2+/NG2+/BLBP+ astrocyte progenitors: a novel component of the neurovascular unit in the developing mouse hippocampus

**DOI:** 10.3389/fncel.2024.1464402

**Published:** 2024-10-17

**Authors:** Shoichiro Omura, Rina Ogawa, Tomomi Kawachi, Aya Ogawa, Yuuki Arai, Natsumi Takayama, Aki Masui, Kumiko Kondo, Hiroki Sugimoto, Hiroshi M. Shinohara, Tokiharu Takahashi, Hideyuki Maeda, Kyoji Ohyama

**Affiliations:** ^1^Department of Histology and Neuroanatomy, Tokyo Medical University, Tokyo, Japan; ^2^Department of Legal Medicine, Osaka University, Suita, Japan

**Keywords:** astrocyte progenitors, olig2, NG2, BLBP, hippocampus, development, neurovascular unit

## Abstract

Astrocytes are key components of the neurovascular unit. While we have recently identified Olig2+ astrocyte progenitors (ASPs) in the developing mouse dentate gyrus (DG), their molecular signature remains incompletely characterized. Here we demonstrate that Olig2+ ASPs predominantly express brain lipid-binding protein (BLBP), while only a small population of them expresses *gfap*-GFP. These Olig2+/BLBP+ ASPs co-express the transcription factors Sox3, Sox9 and the proteoglycan NG2 but not Sox10, a marker for oligodendrocyte progenitors (OLPs). Olig2+ ASPs appear from embryonic day 18 (E18) onwards and decline at postnatal day 14 (P14). Consistent with the proliferation of both Olig2+ and NG2+ glial cells after brain injury, intrauterine intermittent hypoxia (IH) led to an increase in Olig2+/NG2+/BLBP+ ASPs in the postnatal DG. IH also promoted both angiogenesis and vascular coupling of Olig2+/NG2+ ASPs. Our data suggest that IH-induced expression of HIF1a increases Olig2+/NG2+/BLBP+ ASPs in a cell non-autonomous manner. Our data also revealed increased vascular coupling of GFAP+ astrocytes following IH, while the number of GFAP+ astrocytes remains unchanged. Given that BLBP, Olig2 and NG2 are expressed in reactive astrocytes, our findings suggest that Olig2+/NG2+/BLBP+ ASPs represent a subtype of reactive astrocyte progenitors. Furthermore, the enhanced vascular coupling of Olig2+/NG2+/BLBP+ ASPs appears to be an adaptive response to hypoxic brain injury. This study provides new insights into the molecular characteristics of Olig2+/NG2+/BLBP+ ASPs and their potential role in the brain’s response to hypoxic injury, contributing to our understanding of neurovascular unit dynamics in both development and pathological conditions.

## Introduction

Neurovascular unit (NVU) is composed of astrocytes, endothelial cells, pericytes and neurons. Astrocytes, one of the key components of the NVU, contact blood vessels and influence local blood flow by balancing vasoconstriction and vasodilation (Yu et al., 2020). Astrocytes also facilitate vascular angiogenesis through signalling pathways such as VEGF and transforming growth factor β1 (TGFβ1; Virgintino et al., 2003; Welser et al., 2010; Biswas et al., 2017; Siqueira et al., 2018). Astrocytes also contribute to blood brain barrier (BBB) and its permeability, thereby maintaining healthy functioning of the NVU.

The hippocampus plays a crucial role in controlling memory and mental states such as anxiety. In ischaemic injury models, GFAP+ astrocytes in CA1 and the molecular layer of the dentate gyrus (DG) exhibit increased GFAP expression and cellular hypertrophy (Escartin et al., 2021). Increased GFAP expression, i.e., astroglial activation and hypertrophy may affect neurovascular coupling. There is also increasing evidence that activated astrocytes act as an interface between the vasculature and neurons in cognitive disorders (Burda and Sofroniew, 2014; Edison et al., 2018; Price et al., 2018; Diaz Verdugo et al., 2019). It is increasingly important to advance our understanding of the molecular profiles of astrocytes in the hippocampus.

A previous study showed that tenascin C+ astrocyte progenitors (ASPs) differentiate into *gfap* + astrocytes (AS) in the developing mouse hippocampus (Yuasa, 2001). In recent decades, it has become clear that it is crucial to identify molecular markers, such as the expression of transcription factors in ASPs, in order to clarify the molecular and cellular property of astrocytes. However, very little is known about the expression of transcription factors in ASPs in the mouse hippocampus. In the developing mouse neocortex, Sox9-Sox10 antagonism, for instance, governs not only the fate of astrocytes versus oligodendrocytes, but also the classification of astrocytoma versus oligodendroglioma (Glasgow et al., 2014). Mutual antagonism between Sox10 and NFIA regulates diversification of glial lineages and glioma subtypes. NFIA and Sox9 cooperate to control astrogliogenesis in the spinal cord (Kang et al., 2012). Positionally distinct subtypes of white matter astrocytes in the spinal cord, which can be distinguished by the combinatorial expression of Reelin and Slit1, are derived from progenitor domains expressing the homeodomain transcription factors Pax6 and Nkx6.1, respectively (Hochstim et al., 2008). The combinatorial expression of Pax6 and Nkx6.1 governs the positional identity of astrocyte subtypes.

To gain some insight into the development of ASPs in the hippocampus, we focused on the transcription factor Olig2. Olig2 was originally identified to be expressed in early neuronal progenitors such as pMN and oligodendrocyte progenitors (OLPs) (Novitch et al., 2001; Takebayashi et al., 2002). Intriguingly, a previous study also showed that Olig2 is transiently expressed in astrocytes of the postnatal SVZ. More recently, increasing evidence has shown that Olig2 is expressed in an astrocyte subtype of the developing and adult central nervous system (Ohayon et al., 2019; Wang et al., 2021). The Olig2+ ASPs express ALDH1L1 but rarely *gfap* (Ohayon et al., 2019). We have also recently identified Olig2+ ASPs at the molecular layer of the developing mouse DG (Ohyama et al., 2023). However, the marker expression profile of Olig2+ ASP remains unclear. In this study, we have revealed that in the developing mouse hippocampus, Olig2+ ASPs co-express BLBP, Sox3, Sox9 and NG2, but not Sox10, a marker for oligodendrocyte progenitors (OLPs). Given that NG2 glia can act as a subpopulation of reactive astrocytes for tissue repair in brain injury (Bell et al., 2020; Kirdajova et al., 2021), this led us to hypothesise that the Olig2+ ASP might be reactive to brain injury. Indeed, our data show that intrauterine hypoxic injury (IH) increases the number of Olig2+ ASPs and facilitates vascular coupling of the Olig2+ ASPs. Taken together, our data imply that the Olig2 + ASPs may play a role in cerebral vascular homeostasis.

## Materials and methods

### Mice

#### Animals

C57BL6/Ncrl and *gfap*-GFP mice that express GFP under the control of the mouse gfap promoter (Suzuki et al., 2003) were housed under standard conditions (12 h light/dark cycle) at the animal care facility of Tokyo Medical University. All experiments were conducted in accordance with the guidelines of the Institutional Animal Care and Use Committees and conformed to the National Institutes of Health Guide for the Care and Use of Laboratory Animals (NIH Publication No. 80-23) revised in 1996. Every effort was made to minimize the number of animals used and their suffering. Embryos and pups from C57BL6/NCrl and the above *gfap*-GFP transgenic mice were used. The day on which a vaginal plug was found was designated as embryonic day 0.5 (E0.5) and the day of birth was designated as postnatal day 0.5 (P0.5).

### Intrauterine intermittent hypoxia

Pregnant mice were subjected to intermittent hypoxia (IH) on gestational day 11–18 at a rate of 60 cycles/h (nadir 4% O_2_ to peak 21% O_2_) or room air breathing (control) in the same plastic cage placed next to the cage equipped with the IH apparatus for 8 h/d during the light-on period of 12 h. The IH mice had free access to food and water.

### Tissue preparation

Embryos were harvested at E14.5−E18.5 from pregnant mice, and early postnatal day 1–6 (P1-6) mice were anesthetized and sacrificed following approved ethical guidelines. Brain tissues were then isolated and fixed with 4% paraformaldehyde (PFA) in 0.1 M phosphate buffer (PB), pH 7.4, by immersion. P14 mice were transcardially perfused with 15 ml of PBS, followed by 15–30 ml of 4% PFA in 0.1 M PB, pH 7.4, at room temperature for 5–10 min. Brain tissues were isolated and post-fixed by immersion in 4% PFA. After fixation, brains were washed with PBS and immersed in 30% sucrose/0.1 M PB. Forebrains were embedded in OCT compound and stored at −70°C. Cryosections were cut at a thickness of 25 μm.

### Antibodies

The antibodies used in this study are as follows: Guinea pig anti-BLBP IgG (Frontier institute, 1:1000); rabbit anti-BLBP IgG (Millipore, ABN14, 1:1000); rabbit anti-Cyclin D1 (Neomarkers, 9,104-SO, 1:1000); chick anti-GFAP (Millipore, 1:5,000); chick anti-GFP IgY (Abcam ab13970, 1:5,000); rat anti-HIF1a (R&D systems, AF1836, 1:1000); rabbit anti-Iba1 (Wako 019–19,741, 1:1000); rabbit anti-Ki67 antibody (Novocastra, 1:1000); rabbit anti-NG2 IgG (Millipore, 1:1000); rabbit anti-Olig2 IgG (Millipore, AB9610, 1:1,000); goat anti-Olig2 (R&D systems, 1:1,000); rabbit anti-Sox3 serum (gift from T. Edlund, 1:1000); goat anti-Sox9 polyclonal antibody (R&D systems, 1:1,000); goat anti-Sox10 (R&D systems, 1:1000). Alexa 647-Isolectin B4 (Invitrogen, 1:1000) was used to visualize blood vessels.

### Immunohistochemistry

Cryosections were processed for immunohistochemistry as previously described (Ohyama et al., 2023). Briefly, cryosection of the hippocampus were incubated with primary antibodies overnight at 4°C. For some antibody labeling experiments (BLBP, Sox9), antigen retrieval was carried out with Histo VT One (Nacalai, Japan) following manufacturer’s instructions. After three washes with PBS, the sections were incubated with secondary antibodies for 45 min at room temperature. After three washes with PBS, the sections were mounted with Vectashield (Vector lab, CA). Images were taken with a Zeiss LSM700 confocal microscope. In some cases, fluorescence images were digitally zoomed at 0.5x to 2x. Stacks of optical sections (1.8 μm thickness/optical section) were obtained at 0.9 μm increments on the z-axis using an x20 objective. Images were corrected for brightness and contrast and composed using Zeiss Image Browser, ZEN software (Zeiss, Thomwood, NY) and Adobe Photoshop CS6 (San Jose, CA). Mice (*n* = 3–5) were examined for individual experiments and, for quantification of some experiments, 6–15 sections were analyzed for each using Fiji from image J as described previously (Ohyama et al., 2023; Ohyama et al., 2024). AnalyzeSkeleton function was used for quantifying the number of vessel branches. Mean ± SE is given in the results.

## Results

### Molecular signature of Olig2-expressing astrocyte progenitors (ASPs) in the developing mouse hippocampus

**Figure 1:**
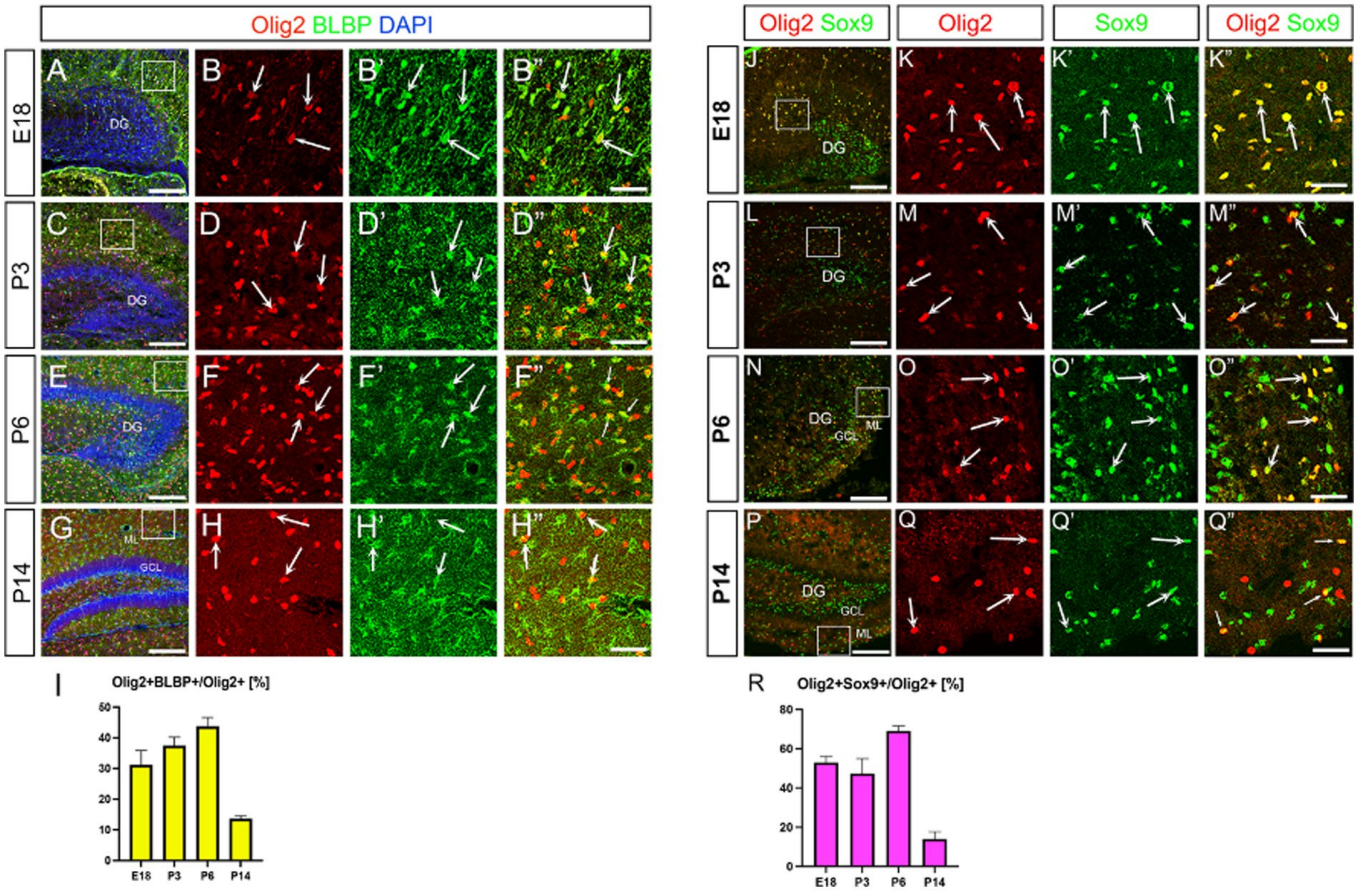
Olig2 expression in BLBP+ and Sox9+ cells in the developing mouse hippocampus. Olig2 expression was detected in BLBP+ cells at embryonic day 18 (E18) (**A**, arrows in **B–B”**, 31.1 ± 4.6%, *n* = 3, 6 sections), postnatal day 3 (P3) (**C**, arrows in **D–D”**, 37.3 ± 2.8%, *n* = 3, 6 sections), P6 (**E**, arrows in **F–F”**, 43.6 ± 2.9%, *n* = 3, 10 sections) and P14 (**G**, arrows in **H–H”**, 13.5 ± 1.0%, *n* = 4, 12 sections). Olig2 is also co-expressed in Sox9+ cells at E18 (**J**, arrows in **K–K”**, 52.9 ± 3.0%, *n* = 4, 12 sections), P3 (**L**, arrows in **M–M”**, 47.3 ± 7.6%, *n* = 4, 11 sections), P6 (**N**, arrows in **O–O”**, 69.0 ± 2.5%, *n* = 4, 11 sections), P14 (**P**, arrows in **Q–Q”**, 13.9 ± 3.6%, *n* = 4, 11 sections). **B–B”, D–D”, F–F”, H–H”. K–K”, M–M”, O–O”, Q–Q”** are magnified images for the boxed area in **A, C, E, G, J, L, N, P**. Quantification data are shown in **I, R**. Scale bars; 200 μm in **A, C, E, G, J, L, N, P**; 50 μm in **B–B”, D–D”, F–F”, H–H”, K–K”, M–M”, O–O”, Q–Q”**.

**Figure 2:**
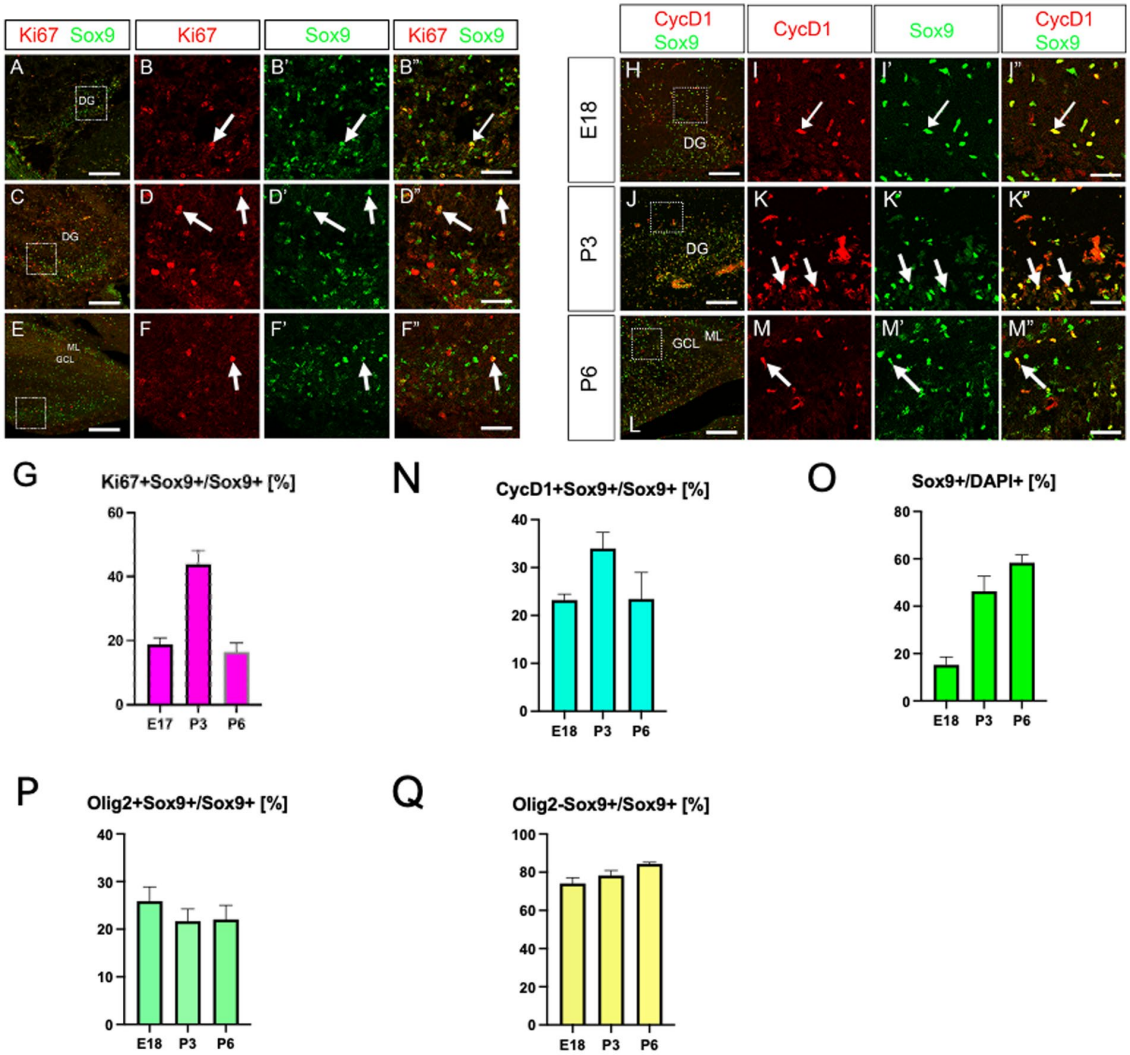
Expression of proliferation markers (Ki67, CycD1) in Sox9+ cells in the developing hippocampus. Ki67 expression was found in Sox9+ cells at E18 (**A**, arrows in **B–B”**, 18.8 ± 2.0%, *n* = 4, 12 sections), P3 (**C**, arrows in **D–D”**, 43.8 ± 4.5%), P6 (**E**, arrows in **F–F”**, 16.5 ± 2.8%, *n* = 3, 6 sections). Similarly, cyclinD1 (CycD1) expression was found in Sox9+ cells at E18 (**H**, arrows in **I–I”**, 23.2 ± 1.2%, *n* = 3, 9 sections), P3 (**J**, arrows in **K–K”**, 33.9 ± 3.3%, *n* = 3, 9 sections), P6 (**L**, arrows in **M–M”**, 23.4 ± 5.5%). Temporal changes in the expression of proliferation markers (Ki67, CycD1) in Sox9+ cells and Sox9+ cells *per se* are shown in **G, N, O**. Quantification graphs for Sox9+ cells/DAPI+ cells, Olig2 + Sox9+ cells /Sox9+ cells, Olig2-Sox9+ cells/Sox9+ cells were shown in **O–Q**: Sox9+ cells/DAPI+ cells [E18 (15.2 ± 3.2%, *n* = 3, 9 sections)], P3 (46.3 ± 6.3%, *n* = 3, 9 sections), P6 (58.3 ± 3.3%, *n* = 3, 9 sections), Olig2 + Sox9+ cells/Sox9+ cells [E18 (25.9 ± 2.9%, *n* = 3, 9 sections)], P3 (21.6 ± 2.5%, *n* = 3, 9 sections), P6 (22.0 ± 2.9%, *n* = 3, 9 sections), and Olig2-Sox9+ cells/Sox9+ cells [E18 (74.1 ± 2.9%, *n* = 3, 9 sections)], P3 (78.32 ± 2.5%, *n* = 3, 9 sections), P6 (84.3 ± 0.8%, *n* = 3, 9 sections). **B–B”, D–D”, F–F”, I–I”, K–K”, M–M”** are magnified images of the boxed area of **A, C, E, H, J, L**. Scale bars; 200 μm in **A, C, E, H, J, L**; 50 μm in **B–B”, D–D”, F–F”, I–I”, K–K”, M–M”**.

We have recently identified Olig2-expressing astrocyte progenitors (Olig2+ ASPs) that co-express *gfap*-GFP (GFP whose expression is driven under the control of the mouse *gfap* promoter) in the developing dentate gyrus (DG) (Suzuki et al., 2003; Ohyama et al., 2023). However, their molecular signature is not fully understood. Consistent with previous studies (Tatsumi et al., 2018), only a small population of Olig2+ cells are GFP+ (12 ± 1%; Supplementary Figure 1). Immunolabelling of Olig2 and GFAP also showed that Olig2+ cells rarely express GFAP (Supplementary Figure 1). Growing bodies of evidence show the existence of GFAP- astrocyte subtypes in the central nervous system (Tatsumi et al., 2018; Ohayon et al., 2019; Wang et al., 2021). These data led us to hypothesise that different astrocyte markers might be expressed in Olig2+ ASPs. A previous study showed that BLBP is barely expressed in the embryonic DG, but begins to be expressed in radial glia-like cells (RGLs) in the early postnatal DG (Matsue et al., 2017). This made BLBP a good candidate to be expressed in postnatal ASPs. We then performed double labelling of Olig2 with BLBP/FABP7, another astrocyte marker in the developing DG. Many Olig2+ cells were found to co-express both BLBP and Sox9 in the molecular layer (ML) (Figure 1). Notably, BLBP+ radial glia-like cells (RGLs) in the subgranular zone (SGZ) did not co-express Olig2 (Figure 1). This confirms our view that gfap-GFP+ RGLs at the SGZ do not express Olig2 (Ohyama et al., 2023). Interestingly, Olig2+/BLBP+/Sox9+ ASPs increase during embryonic day 18 (E18)-postnatal day 6 (P6), but decrease at P14 (Figures 1I,R). Consistent with the Olig2+ cells being ASPs, Olig2+/Sox10− cells were found, in addition to Olig2+/Sox10+ oligodendrocyte progenitors (OLP; Supplementary Figure 2). Concomitant with the decreased ratio of Olig2+ ASPs vs. Olig2+ cells, Olig2 + Sox10+ OLPs/Olig2+ cells increase at P14. These data suggest that the ratio of Olig2+/Sox9+ ASPs vs. Olig2+/Sox10+ cells among Olig2+ cells is balanced. Our data also show that the Sox9+ cells also express both Ki67 and cyclin D1 (CycD1), suggesting that the Olig2+/Sox9+ cells are proliferative ASPs (Figures 2A–N). Consistent with this, Sox9+ cells increased over time during E18-P6. They are comprised of Olig2+/Sox9+ ASPs at approximately 20% and Olig2+/Sox9+ radial glia-like cells (RGLs) at 80% during E18-P6 (Figures 2O–Q).

**Figure 3:**
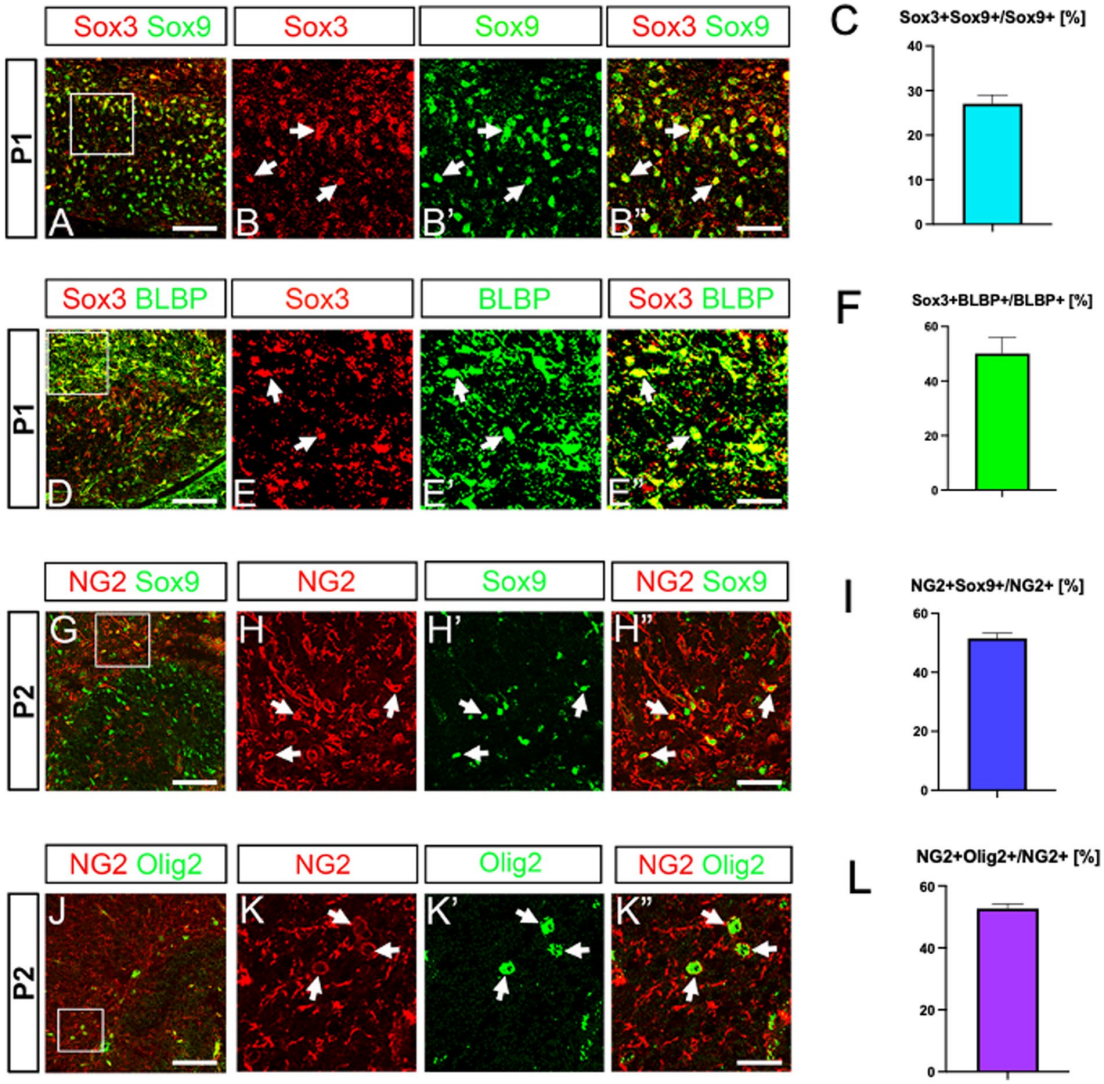
Co-expression of Sox3, BLBP and NG2 in Sox9+ ASPs. Sox3 is expressed in Sox9+/BLBP+ ASPs (**A**, arrows in **B–B”, D**, arrows in **E–E”**). NG2 expression was found in Sox9+ ASPs as well as in Olig2+ cells (**G**, arrows in **H–H”, J**, arrows in **K–K”**). Quantification analyses show Sox3 + Sox9+ cells/Sox9+ cells (27.0 ± 1.9%, *n* = 3, 9 sections), Sox3 + BLBP+ cells/BLBP+ cells (50.0 ± 5.9%, *n* = 3, 8 sections), NG2 + Sox9+ cells/NG2+ cells (51.5 ± 1.8%, *n* = 4, 12 sections), NG2 + Olig2+ cells/NG2+ cells (52.6 ± 1.5%, *n* = 5, 14 sections) (**C, F, I, L**). Scale bars; 200 μm in **A, D, G, J**; 50 μm in **B–B”, E–E”, H–H”, K–K”.**

A previous study showed that Sox3 and Sox9 are co-expressed in glial progenitors, but not in neuronal progenitors (Klum et al., 2018). The authors also showed that astrocytes are Sox3-/Sox9+, whereas ASPs are Sox3+/Sox9+. Our data showed that Olig2+/Sox9+/BLBP+ cells express Sox3 (Figures 3A–F). These data support the idea that the Olig2+/Sox9+/BLBP+ cells are ASPs. NG2/cspg4 is known to be expressed mainly in OLPs, but it is also expressed in some astrocytes (Zhu et al., 2008; Janeckova et al., 2024). Thus, we carried out double labelling of NG2 and Sox9. NG2+/Sox9+ ASPs were found in the developing DG (Figures 3G–I). Co-expression of NG2 and Olig2 was also found (Figures 3J–L). However, BLBP+ ASPs did not co-express Sox10 (Supplementary Figures S2K,L–L”). Taken together, our data suggest that there are at least two distinct populations of Olig2+ progenitors in the developing mouse DG, namely ASPs (Olig2+/Sox3+/Sox9+/BLBP+/NG2+) and OLPs (Olig2+/Sox10+/NG2+).

#### Intrauterine intermittent hypoxia facilitates the vascular coupling of Olig2+ ASPs

**Figure 4:**
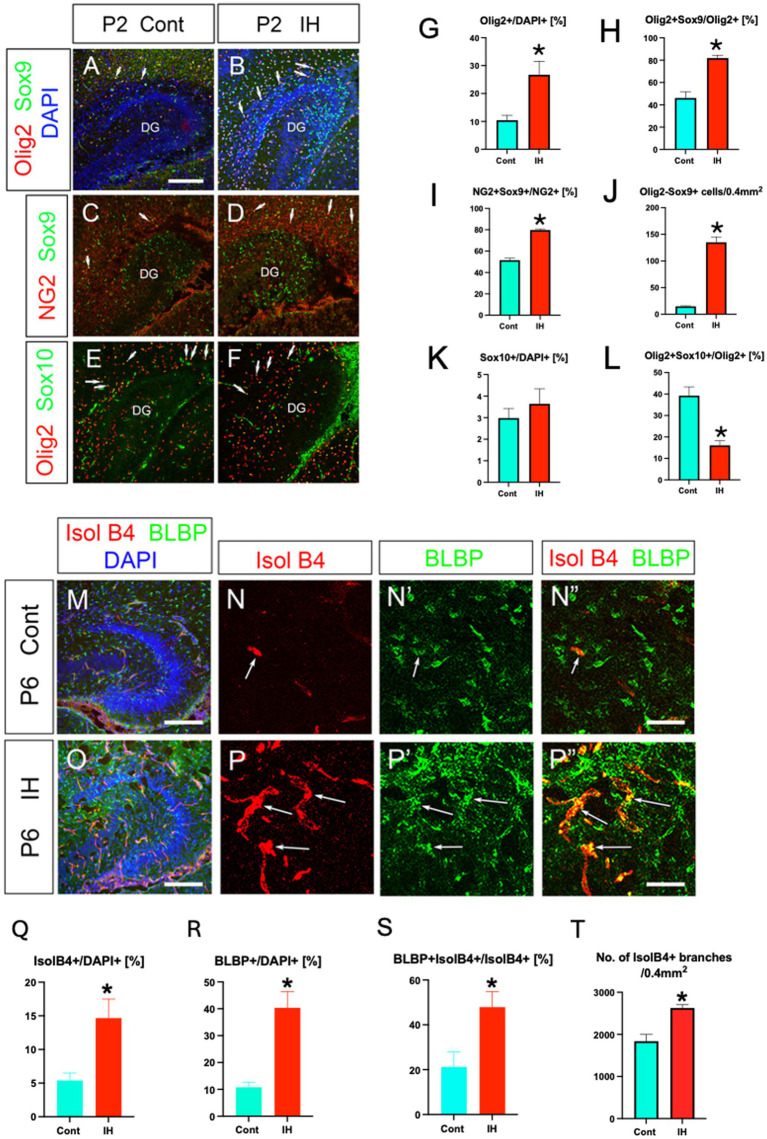
IH increases Olig2+ ASPs and facilitates their vascular coupling. Olig2+ cells/DAPI+ cells increased under intrauterine hypoxia (IH) at P2 (**A, B, G**) [Control (Cont), 10.4 ± 1.7%, *n* = 3, 6 sections] vs. IH (26.7 ± 4.8%, *n* = 3, 8 sections), two-tailed *t*-test, *p* = 0.0219). Olig2+/Sox9+ ASPs vs. Olig2+ cells were increased in the IH model (**A, B, H**) [Cont (46 ± 5.6%, *n* = 5, 15 sections] vs. IH (81.8 ± 2.5%, *n* = 4, 13 sections), two-tailed *t*-test, *p* < 0.0001 (**A, B, H**). Consistent with this, NG2+/Sox9+ ASPs vs. NG2+ cells were increased under the IH condition [**C, D, I**, Cont (51.5 ± 1.8%, *n* = 4, 12 sections), vs. IH (79.7 ± 1.0%, *n* = 4, 12 sections), two-tailed *t*-test, *p* < 0.0001]. RGLs (Olig2-Sox9+) were significantly increased in the IH model [**J**, Cont (14.9 ± 0.9 cells, *n* = 5, 15 sections), IH (135.1 ± 10.2 cells, *n* = 4, 13 sections), two-tailed *t*-test, *p* < 0.0001]. The number of Sox10+ cells/DAPI did not change between Cont (K, 2.9 ± 0.4%, *n* = 3, 8 sections) and IH (**K**, 3.6 ± 0.6%, *n* = 3, 9 sections). Olig2 + Sox10+ OLPs/Olig2+ cells were decreased (**L**) (Cont (39.2 ± 4.0%, *n* = 3, 8 sections), 16.8 ± 2.1% (IH, *n* = 3, 9 sections), two-tailed *t*-test, *p* = 0.0001). IH increased the density of both IsolB4 + blood vessels at P6 (**M, N, O, P, Q**) [Cont (5.3 ± 1.0 ± %, *n* = 3, 7 sections) vs. IH (14.6 ± 2.8%, *n* = 3, 8 sections), two-tailed *t*-test, *p* = 0.0124] and BLBP+ ASPs (**N’, P’, R**) [Cont (10.7 ± 1.9%, *n* = 3, 6 sections) vs. IH (40.3 ± 6.1%, *n* = 3, 6 sections), two-tailed *t*-test, *p* = 0.0009] (**G1–G4, H1–H4, I–K**). Many more BLBP+ ASPs were closely associated with the blood vessels after IH (**N”, P”, S**, see Supplementary Figure S3) [Cont (21.2 ± 6.6%, *n* = 3, 7 sections) vs. IH (47.8 ± 6.9%, *n* = 3, 8 sections), two-tailed *t*-test, *p* = 0.0163]. No. of IsolB4+ vessel branches was increased under the IH condition **(T)** [Cont (1,838 ± 161.5, *n* = 3, 9 sections) vs. IH (2,627 ± 76.1, *n* = 4, 9 sections), two-tailed *t*-test, *p* = 0.0004]. Scale bars; 200 μm in **A–F, M, O**; 50 μm in **N–N”, P–P”.**

Brain injury such as hypoxia leads to an increase in Olig2+ cells (Dizon et al., 2010; Allan et al., 2021). NG2 is known to be expressed by reactive astrocytes after brain injury (Honsa et al., 2016). To gain some insight into the property of Olig2+ ASPs, we next examined the response of Olig2+ ASPs to hypoxic injury. To address this question, we used an intrauterine hypoxia paradigm that mimics sleep apnea syndrome during pregnancy (Johnson et al., 2018), as the Olig2+ ASPs start to develop in the DG during the perinatal period. Our data showed that Olig2+ cells/DAPI+ cells increased, and that the ratio of Olig2+/Sox9+ ASPs/Olig2+ cells was also increased, at postnatal day (P2; Figures 4A,B,G,H). → Olig2+/Sox9+ ASPs vs. Olig2+ cells was also increased, at postnatal day 2 (P2) (Figures 4A,B,G,H). Similarly, the ratio of NG2 + Sox9+ cells vs. NG2+ cells increased in the IH model at P2 (Figures 4C,D,I). Given that RGLs are Olig2-/Sox9+ (Ohyama et al., 2023), our data showed that Olig2-/Sox9+ RGLs are increased (Figure 4J). While the number of Sox10+ OPCs did not change in the IH model, the ratio of Olig2+/Sox10+ OLPs vs. Olig2+ cells was decreased (Figures 4E,F,K,L). Together, IH increases both RGLs and Olig2+ ASPs, whereas the ratio of OLPs vs. Olig2+ cells decreased, controlling the balance of ASPs and OLPs during development.

Isolectin B4 (IsolB4) + blood vessels became more dense under the IH condition at P6 (Figures 4M–Q), and many more BLBP+ ASPs were closely associated with the blood vessels (Figures 4N’,N”,P’,P”,R,S). Given that IsolB4 labels not only endothelial cells but also immature astrocytes located adjacent to the tips of developing blood vessels (Chung and Cho, 2008), BLBP+ ASPs are closely associated with newly generated blood vessels (Supplementary Figure S3). Our data also showed that the number of vessel branches increased in the IH model, compared to the control (Cont; Figure 4T). These data suggest that IH facilitated the vascular coupling of Olig2+/NG2+/BLBP+ ASPs, a novel component of NVU.

**Figure 5:**
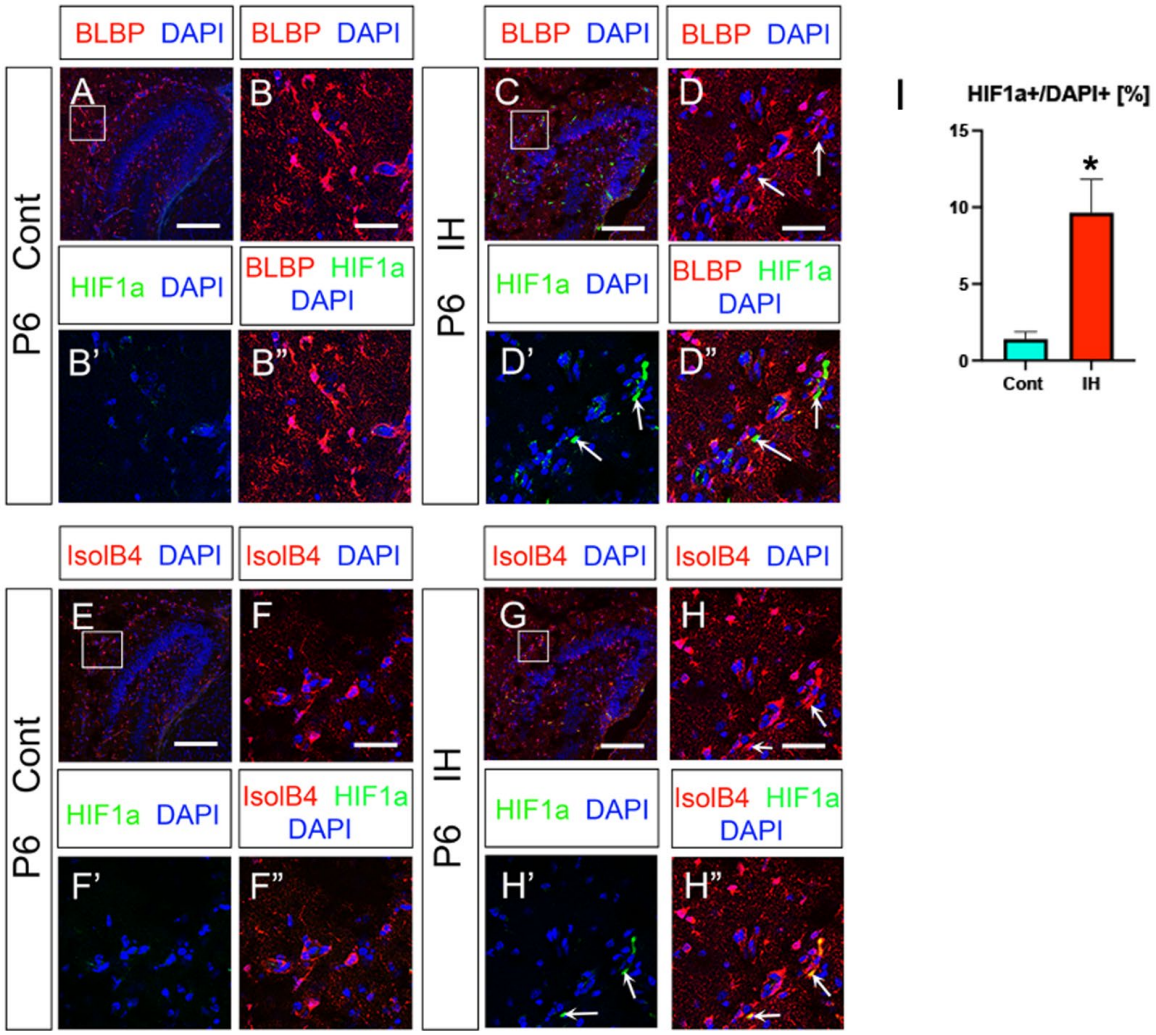
IH increases HIF1a + endothelial cells. IH increased HIF1a + cells/DAPI+cells (%) (**A, B–B”, C**, arrows in **D–D”, I**, Cont (1.3 ± 0.4%, *n* = 3, 9 sections) vs. IH (9.6 ± 2.1%, *n* = 3, 9 sections), two-tailed *t*-test, *p* = 0.0019). The HIF1a + cells are not BLBP+ ASPs at P6 (**C, D–D”**). IH-induced HIF1a + cells are IsolB4+ endothelial cells (**G**, arrows in **H–H”**, *n* = 3, 9 sections for Cont and IH, respectively). Scale bars; 200 μm in **A, C, E, G**; 50 μm in **B–B”, D–D”, F–F”, H–H”**.

Hypoxic conditions induce the transcription factor HIF1a (Allan et al., 2021). In the present study, we observed an increase in HIF1a + cells in the IH model compared to controls (Figures 5A, B–B”,C,D–D”,E, F–F”,G,H–H”,I). Intriguingly, HIF1a expression was found in IsolB4+ endothelial cells but not BLBP+ ASPs (arrows in Figures 5D–D,”H–H”). These data suggest that endothelial HIF1a expression promotes the expansion of Olig2+ ASPs.

**Figure 6:**
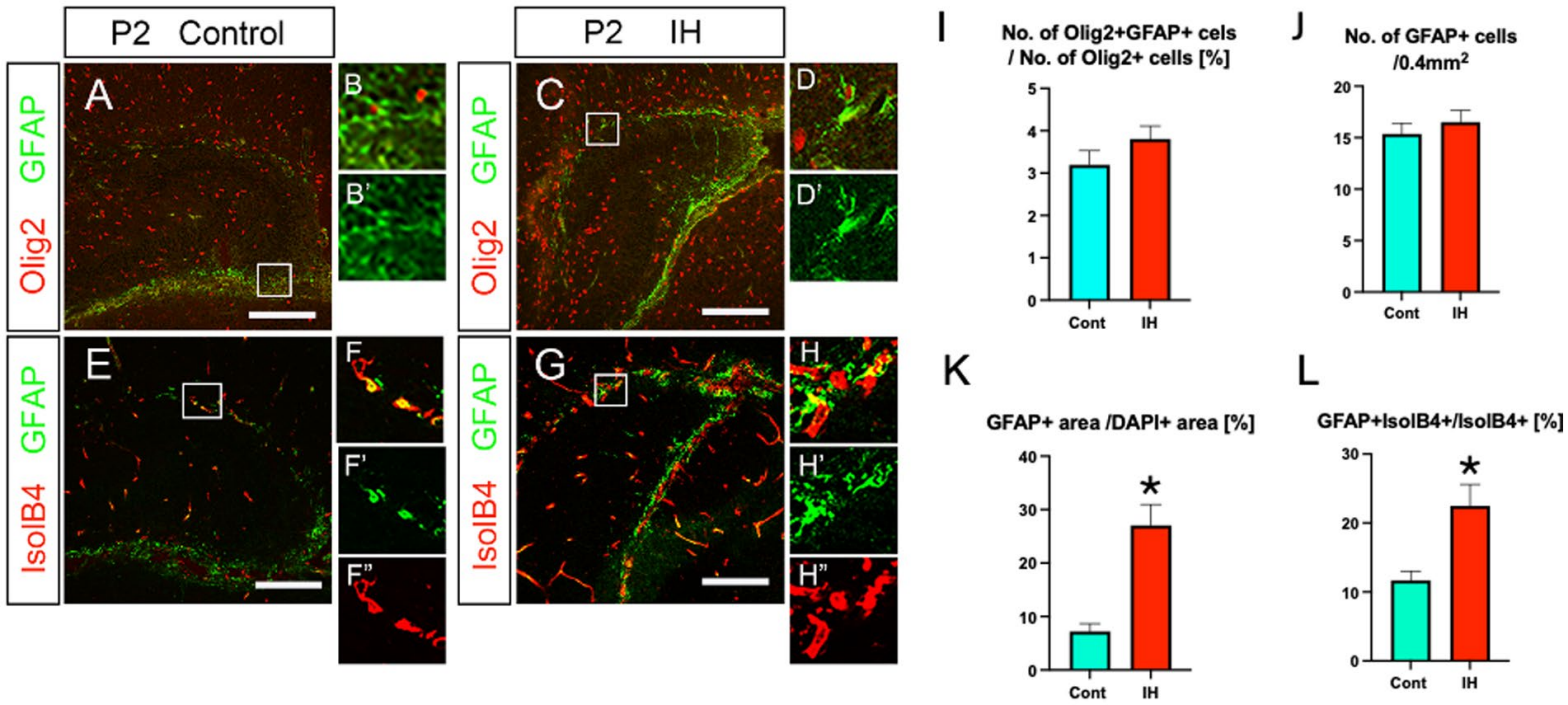
IH increases the area of both GFAP+ processes and their coupling with IsolB4+ blood vessels, while neither the number of Olig2+/GFAP+ astrocytes nor that of GFAP+ astrocytes changes in IH. The ratio of Olig2 + GFAP+ astrocytes/Olig2+ cells did not change in IH at P2 [Cont (3.1 ± 0.3%, *n* = 3, 9 sections) vs. IH (3.8 ± 0.3%, *n* = 5 for IH, 14 sections), two-tailed unpaired *t*-test, *p* = 0.2002]. The number of GFAP+ astrocytes did not change in IH at P2 [Cont (15.3 ± 1.0%, *n* = 4, 11 sections) vs. IH (16.5 ± 1.1%, *n* = 5, 14 sections), two-tailed unpaired *t*-test, *p* = 0.4855] (**A, B, B’, C, D, D’, I, J**). GFAP+ area/DAPI+ area (%) increased in the IH model [Cont (7.2 ± 1.4%, *n* = 4, 12 sections) vs. IH (27.6 ± 3.8%, *n* = 5, 14 sections), two-tailed unpaired *t*-test, *p* = 0.0001] (**A, C, E, G, K**). Vascular coupling of GFAP+ astrocytes was also increased [Cont (11.6 ± 1.3%, *n* = 3, 8 sections) vs. IH (22.4 ± 3.1%, *n* = 4, 12 sections), two-tailed unpaired *t*-test, *p* = 0.0146] (**E, F–F”, G, H–H”, L**). Scale bars; 200 μm in **A, C, E, G**.

We next asked the question of whether the increased Olig2+ ASPs are unique response to IH or other astrocyte subtypes such as GFAP+ astrocytes also respond to the IH. Our data show that the number of Olig2 + GFAP+ astrocytes and total GFAP+ astrocytes did not change in the IH model (Figures 6A,B–B’,C,D–D’,E, F–F”,G,H–H”,I,J). However, GFAP+ area was increased in the IH model (Figure 6K). In addition, GFAP+/IsolB4+ area also increased. These data suggest that GFAP+ astrocytes respond to IH by promoting vascular coupling but not increasing the cell number. This contrasts with the response of Olig2+ ASPs to IH, which involves both an increase in cell number and vascular coupling.

**Figure 7:**
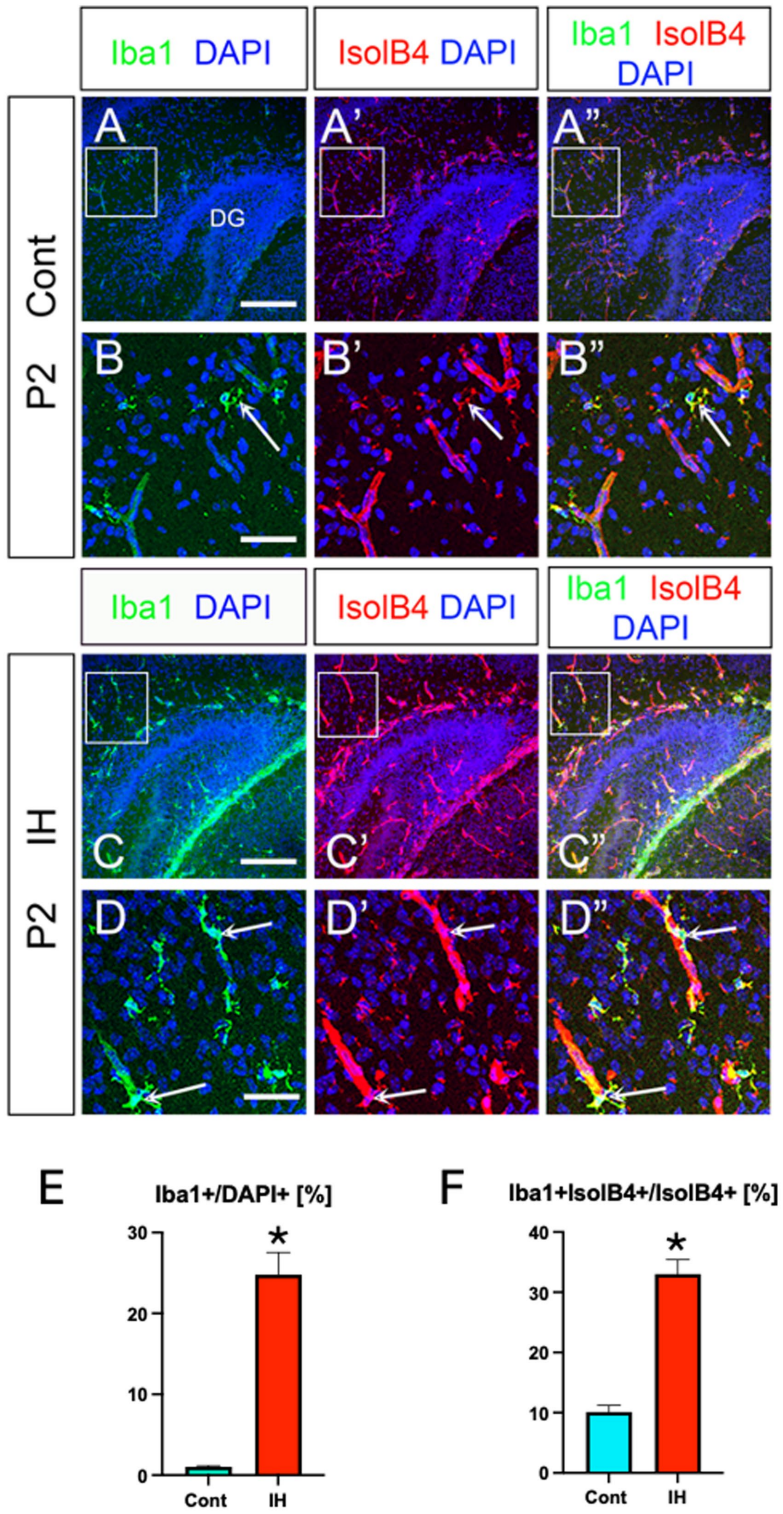
IH increases Iba1+ microglia and their vascular coupling. Iba1+ microglia and their vascular coupling were increased in IH at P2 (**A–A”**, arrows in **B-B”, C–C”,** arrows in **D–D”, E, F**). Iba1+/DAPI+ [Cont (1.0 ± 0.1%, *n* = 3, 9 sections) vs. IH (24.7 ± 2.7%, *n* = 3, 9 sections), two-tailed *t*-test, *p* < 0.0001], Iba1 + IsolB4+ area /IsolB4+ area [Cont (10.1 ± 1.0%, *n* = 3, 9 sections) vs. IH (33.0 ± 2.4%, *n* = 3, 9 sections), two-tailed *t*-test, *p* < 0.0001]. Scale bars; 200 μm in **A–A”, C–C”**; 50 μm in **B–B”, D–D”**.

Lastly, we monitored Iba1+ microglia in the IH model. Both Iba1+ microglia and their association with IsolB4+ blood vessels increased (Figures 7A–A”,B–B”,C–C”,D–D”,E, F). Taken together, our data show that IH increases Olig2+/NG2+/BLBP+ ASPs and microglia in their number and promotes vascular coupling, whereas GFAP+ astrocytes increase their vascular coupling without changing cell number. In contrast, the ratio of OLPs vs. ASPs decreased in the IH condition. In summary, our study highlights the distinct adaptive responses of glial cells in the developing mouse hippocampus.

## Discussion

### Distinct marker expression profile of Olig2+ ASPs in the hippocampus

In this study, we have characterized the unique marker expression profile of Olig2+ ASPs in the hippocampus. Our findings reveal that Olig2+/BLBP+/Sox9+ ASPs share several markers with OLPs, including NG2 and Olig2. However, a key distinction is their lack of Sox10 expression, which is replaced by the glial progenitor marker Sox3. Notably, we observed that Sox3 is co-expressed with Sox9 but not Sox10 in Olig2+ ASPs, aligning with previous findings that Sox3 and Sox9 are co-expressed in ASPs but absent in differentiated astrocytes (Holmberg et al., 2008). The mechanism underlying this Sox10 suppression in Olig2+ ASPs remains to be elucidated.

Our results, consistent with previous studies (Tatsumi et al., 2018; Ohayon et al., 2019; Wang et al., 2021), demonstrate that Olig2+ ASPs rarely co-express GFAP, although some overlap with *gfap*-GFP+ cells was observed (Supplementary Figure 1). This suggests that the fate determination between ASPs and OLPs is not solely dependent on Olig2 expression. Furthermore, our data indicate that Olig2 expression is specific to ASPs or OLPs, but not RGLs in the SGZ. This conclusion is supported by our observations that neither *gfap*-GFP+ RGLs nor BLBP+ RGLs at the SGZ express Olig2 (Figure 1), corroborating our recent study (Ohyama et al., 2023).

The fate switching mechanism between ASPs and OLPs appears to be regulated by NFIA-Sox10 antagonism (Glasgow et al., 2014). In this context, NFIA protein directly interacts with Sox10, mutually repressing each other’s transcriptional functions. Conversely, NFIA binds to Sox9, cooperatively activating ASP-specific genes such as Mmd2 (Kang et al., 2012). These physical interactions between NFIA and either Sox9 or Sox10 likely play a crucial role in determining ASP versus OLP fate in the developing hippocampus.

### Olig2+/NG2+/BLBP+ ASPs exhibit distinct responses to intermittent hypoxia

Previous research has shown that various cell types, including Olig2+ cells, NG2+ cells, and pericytes, increase in response to brain injury or hypoxic conditions (Buffo et al., 2005; Jablonska et al., 2012; Keskin et al., 2015). Hypoxia-inducible factor (HIF) has been implicated in mediating NG2 and Sox9 expression (Ampofo et al., 2017; Amarilio et al., 2007). Interestingly, while hypoxia increases Olig2+ cells via HIF1a, it simultaneously downregulates the OLP marker, Sox10, thereby inhibiting oligodendrocyte differentiation (Dizon et al., 2010; Allan et al., 2021).

Our study provides new insights into the specific response of Olig2+/NG2+/BLBP+ ASPs to intermittent hypoxia (IH). We found that IH induced HIF1a expression in endothelial cells, leading to two significant outcomes: an increase in Olig2+/Sox9+/BLBP+ ASPs and a decrease in the ratio of Olig2+/Sox10+ OLPs to Olig2+ ASPs (Figure 4). Moreover, IH enhanced the vascular coupling of Olig2+/NG2+/BLBP+ ASPs. These data are particularly interesting in light of previous research showing that pERK-HIF1α-p21 signaling operates in tip cells and controls sprouting and arrest in angiogenesis (Pontes-Quero et al., 2019). It will be important to explore this pathway using our IH model in future studies.

These findings suggest that Olig2+/NG2+/BLBP+ ASPs may represent a developmental origin of a subpopulation of reactive astrocytes in the developing hippocampus. Our recent work (Ohyama et al., 2023) demonstrated that Olig2+ ASPs express pSmad3, a marker for anti-inflammatory A2 astrocytes, indicating a potential beneficial role in brain tissue homeostasis.

Interestingly, we observed distinct responses to IH between Olig2+/NG2+/BLBP+ ASPs and GFAP+ astrocytes. While IH increased the number and vascular coupling of Olig2+/NG2+/BLBP+ ASPs, it induced hypertrophy and enhanced vascular coupling of GFAP+ astrocytes without altering their cell number (Figure 6). This differential response suggests unique roles for these cell populations in adapting to hypoxic conditions.

### Developmental origins and regulatory mechanisms of Olig2+ ASPs

While we have characterized the marker expression profile of Olig2+ ASPs (BLBP+, Sox3+, Sox9+, NG2+, and Sox10−), their developmental origin and the signals controlling their specification require further investigation. A previous study (Yuasa, 2001), suggested that there are two distinct migratory pathways for ASPs: one originating from the dentate notch (DN), the origin of the dentate gyrus (DG), and the other from the prospective CA1 region. The distribution patterns of Olig2+ ASPs in the molecular layer of the DG and CA1 suggest they may originate from both sources. However, the relatively lower number of Olig2+ cells in the DN, hilus, and DG compared to prospective CA1 implies that the majority of Olig2+ ASPs may arise from the ventricular zone of prospective CA1.

Several signaling pathways may be involved in regulating the molecular signature of Olig2+ ASPs. Retinoic acid (RA) signaling is a potential candidate, as Raldh2, an RA synthetic enzyme, is expressed in the hippocampal fissure and meninges surrounding the developing DG (Niederreither et al., 2002). RA signaling has been shown to induce HIF1a expression in the adult mouse DG (Mishra et al., 2018). Wnt signaling may also play a role, as Lef1, a downstream target of Wnt, is expressed in the developing DG (Galceran et al., 2000), suggesting a potential involvement in the development of Olig2+ ASPs derived from the DN.

Our recent findings implicate TGFβ signaling in Olig2+ ASP development. We demonstrated that pSmad3, a downstream effector of TGFβ, is expressed in Olig2+ ASPs (Ohyama et al., 2023). Additionally, TGFβ regulates NG2 expression (Ampofo et al., 2017), further supporting its role in Olig2+ ASP development. Notch signaling is another potential regulator, as Smad3 interacts with NICD, an intracellular domain of Notch, to maintain the undifferentiated state of neural progenitors. Notch activity requires Sox3 (Holmberg et al., 2008) and positively regulates BLBP expression (Anthony et al., 2005).

The interplay between these signaling pathways, particularly the cross-talk between Notch and TGFβ, may contribute to establishing the unique marker expression profile of Olig2+ ASPs. Further research is needed to elucidate the precise mechanisms governing the identity of these cells.

In conclusion, we have identified Olig2+/NG2+/BLBP+ ASPs as a novel component of the neurovascular unit (NVU) that responds distinctively to hypoxic injury. These cells represent a unique population with characteristics distinct from both oligodendrocyte progenitors and mature astrocytes. Their response to intermittent hypoxia suggests a potential role in adapting to and potentially mitigating the effects of hypoxic conditions in the developing hippocampus.

Future research should focus on elucidating the specific roles of these ASPs in vascular coupling, their contribution to maintaining the blood–brain barrier (BBB), and their potential involvement in regulating blood flow in the hippocampus.

## Data Availability

The original contributions presented in the study are included in the article/Supplementary material, further inquiries can be directed to the corresponding author.
