# Supplementary figures and images for: Olig2+/NG2+/BLBP+ astrocyte progenitors: a novel component of the neurovascular unit in the developing mouse hippocampus
Source: Front Cell Neurosci. 2024 Oct 17;18:1464402. doi: 10.3389/fncel.2024.1464402 (PMC11524929; doi:10.3389/fncel.2024.1464402)

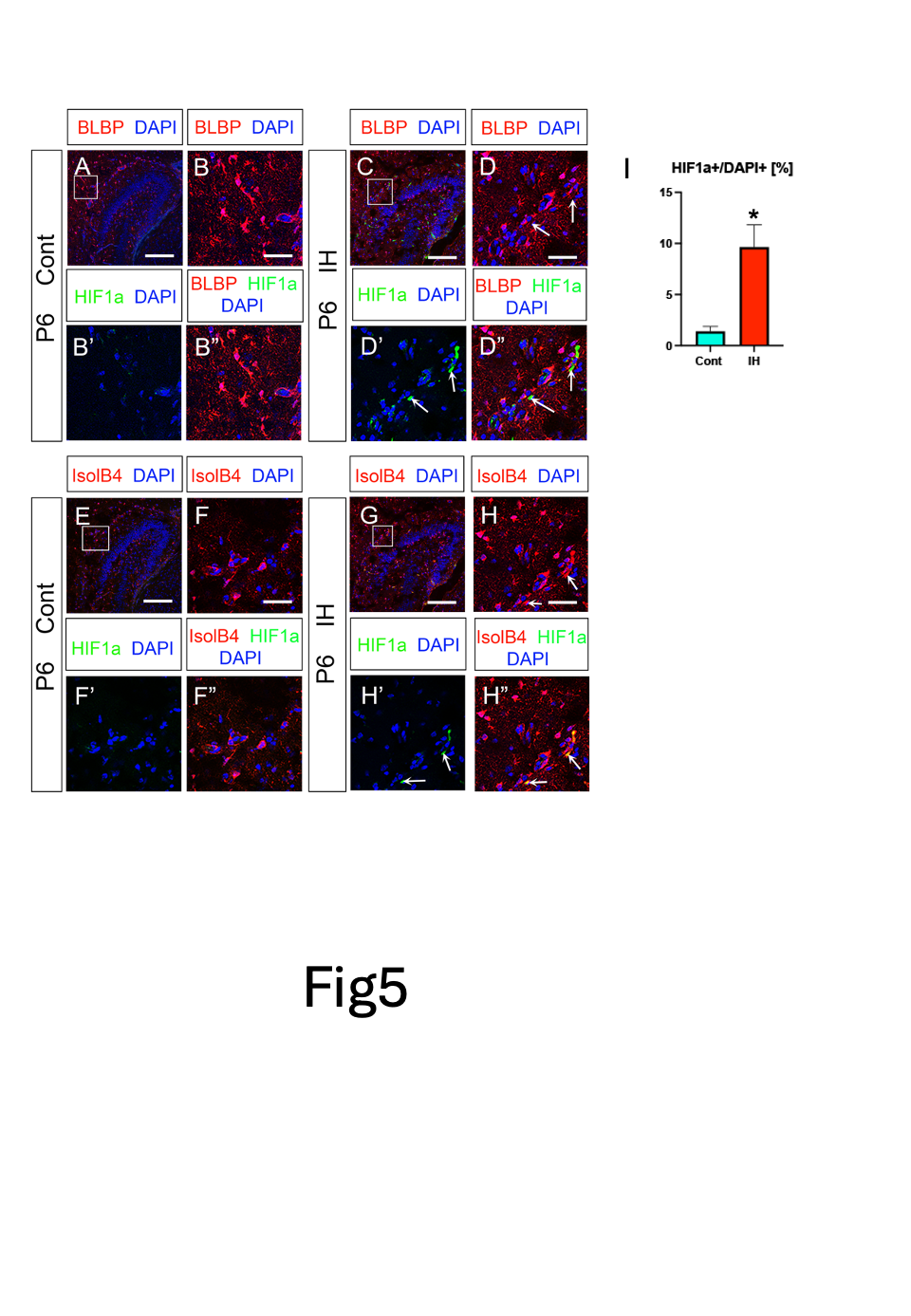

Supplement: Supplementary file 1 [file Image_1.TIFF]

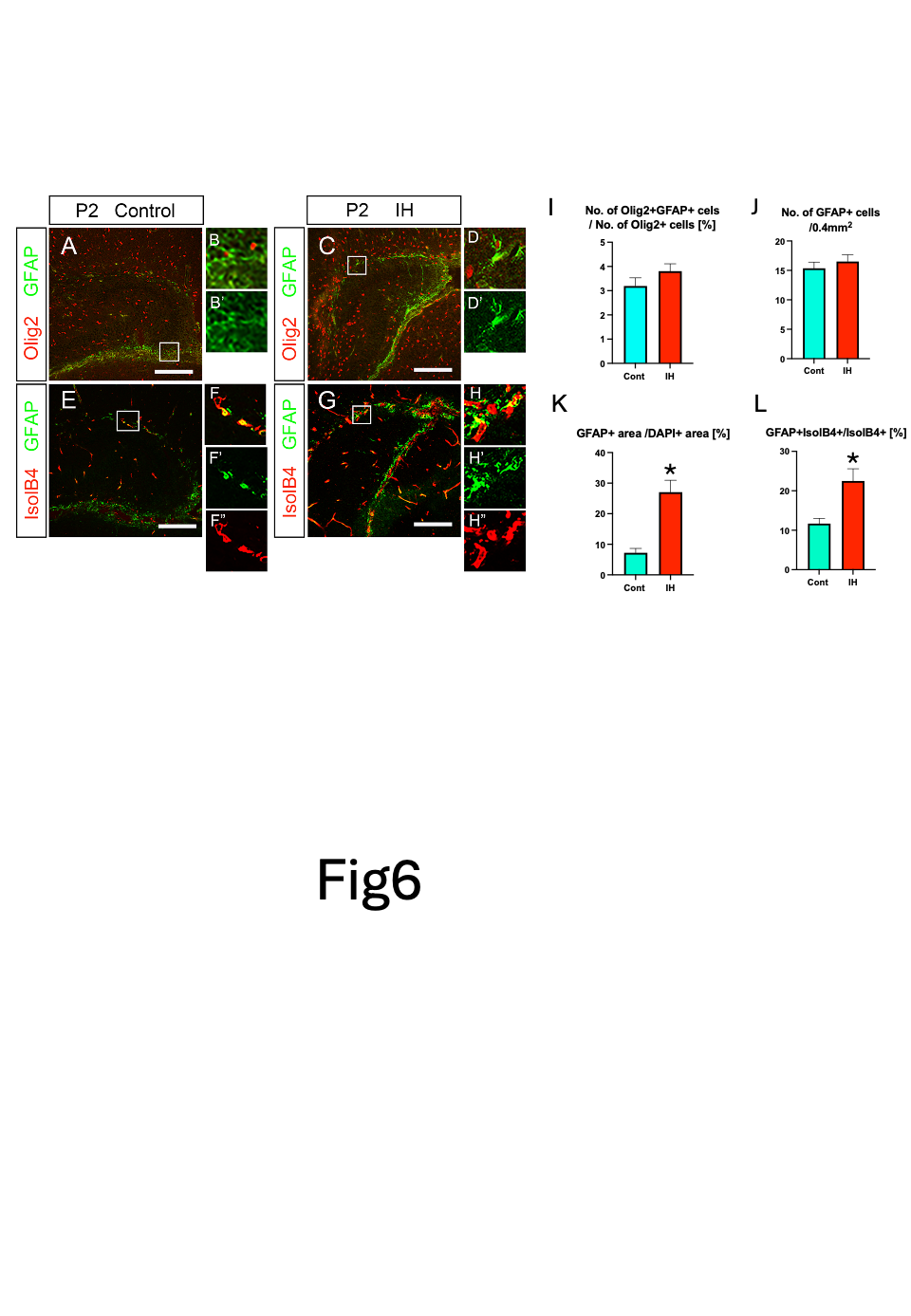

Supplement: Supplementary file 2 [file Image_2.TIFF]

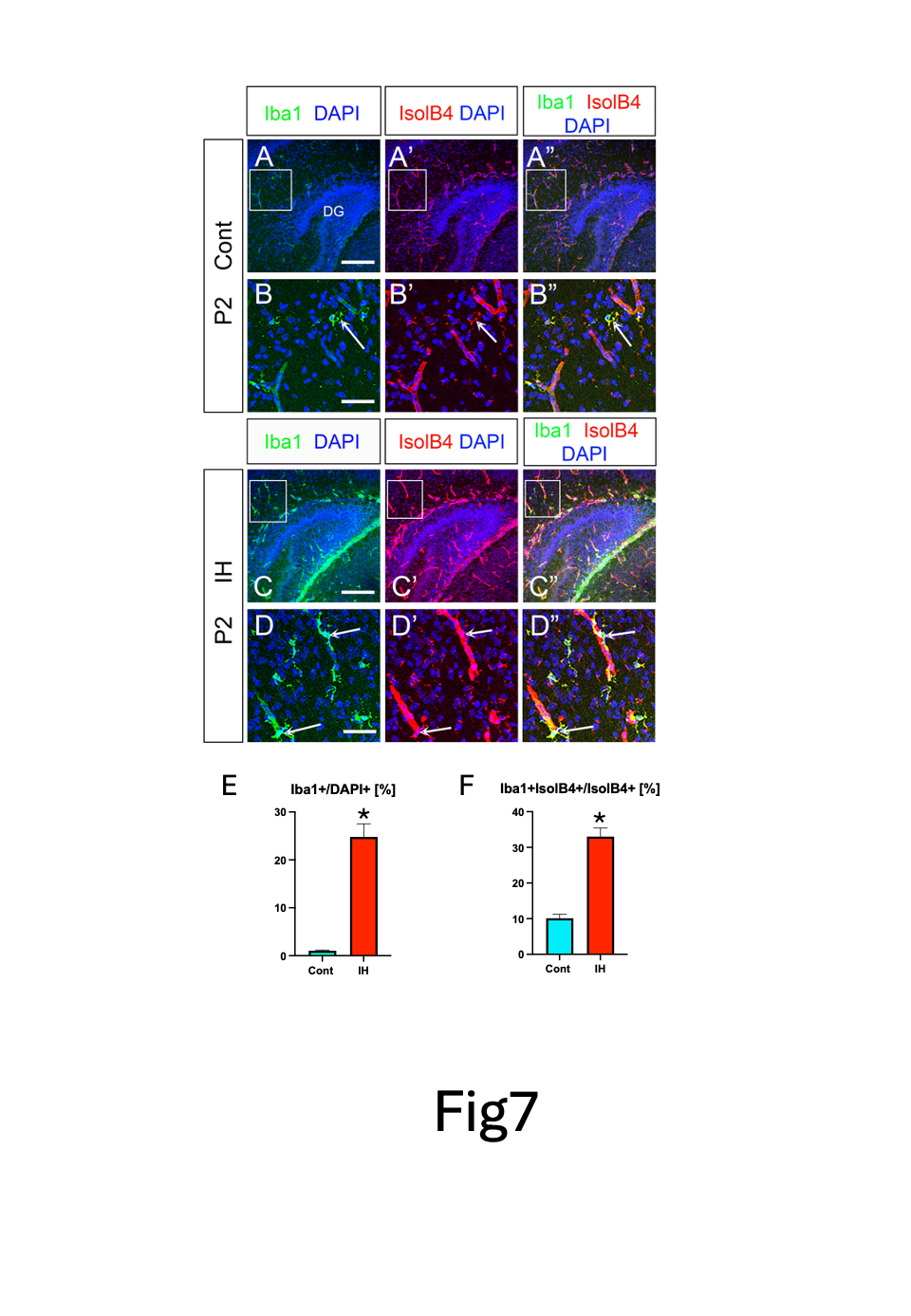

Supplement: Supplementary file 3 [file Image_3.TIFF]

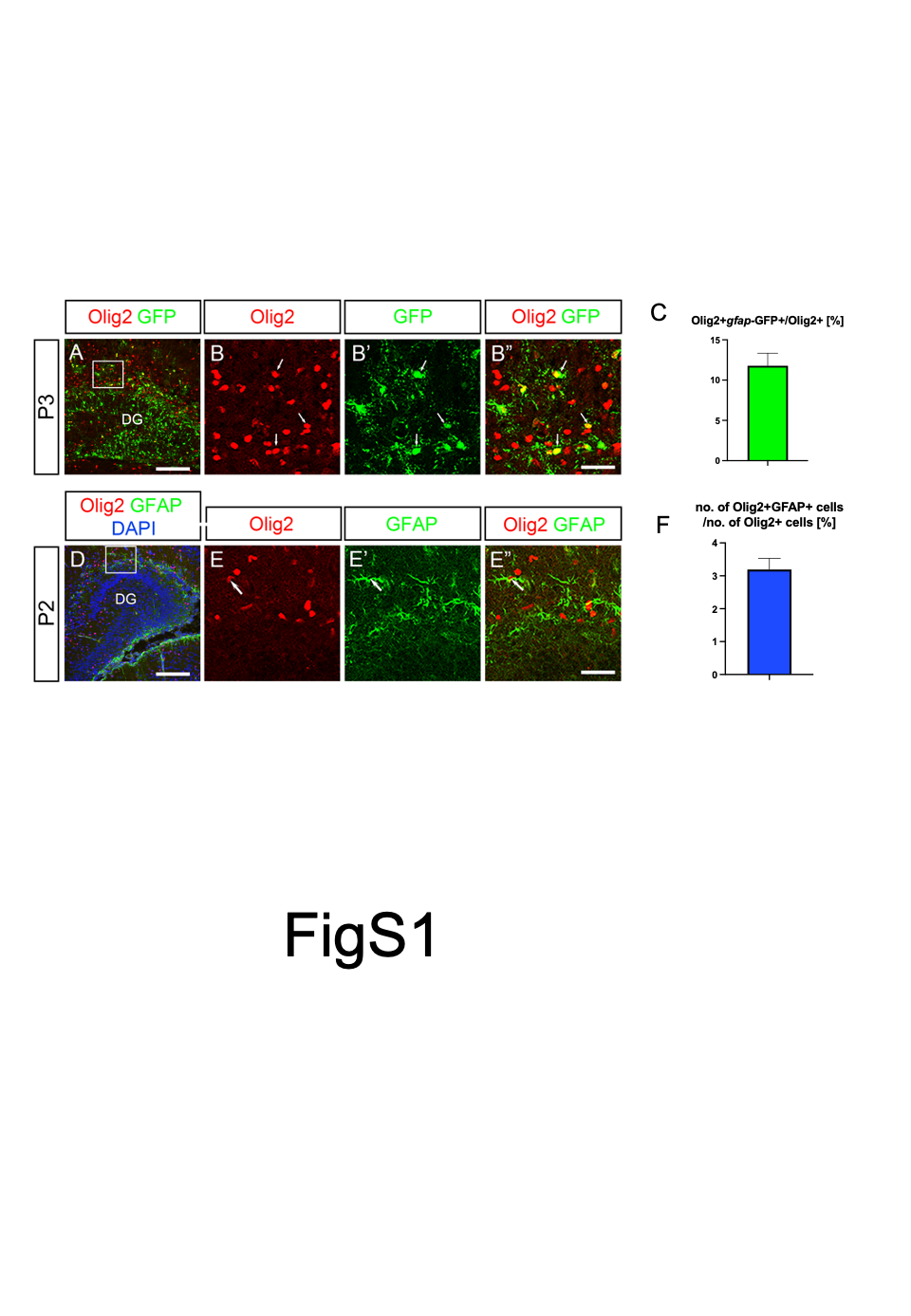

Supplement: Supplementary file 4 [file Image_4.TIFF]

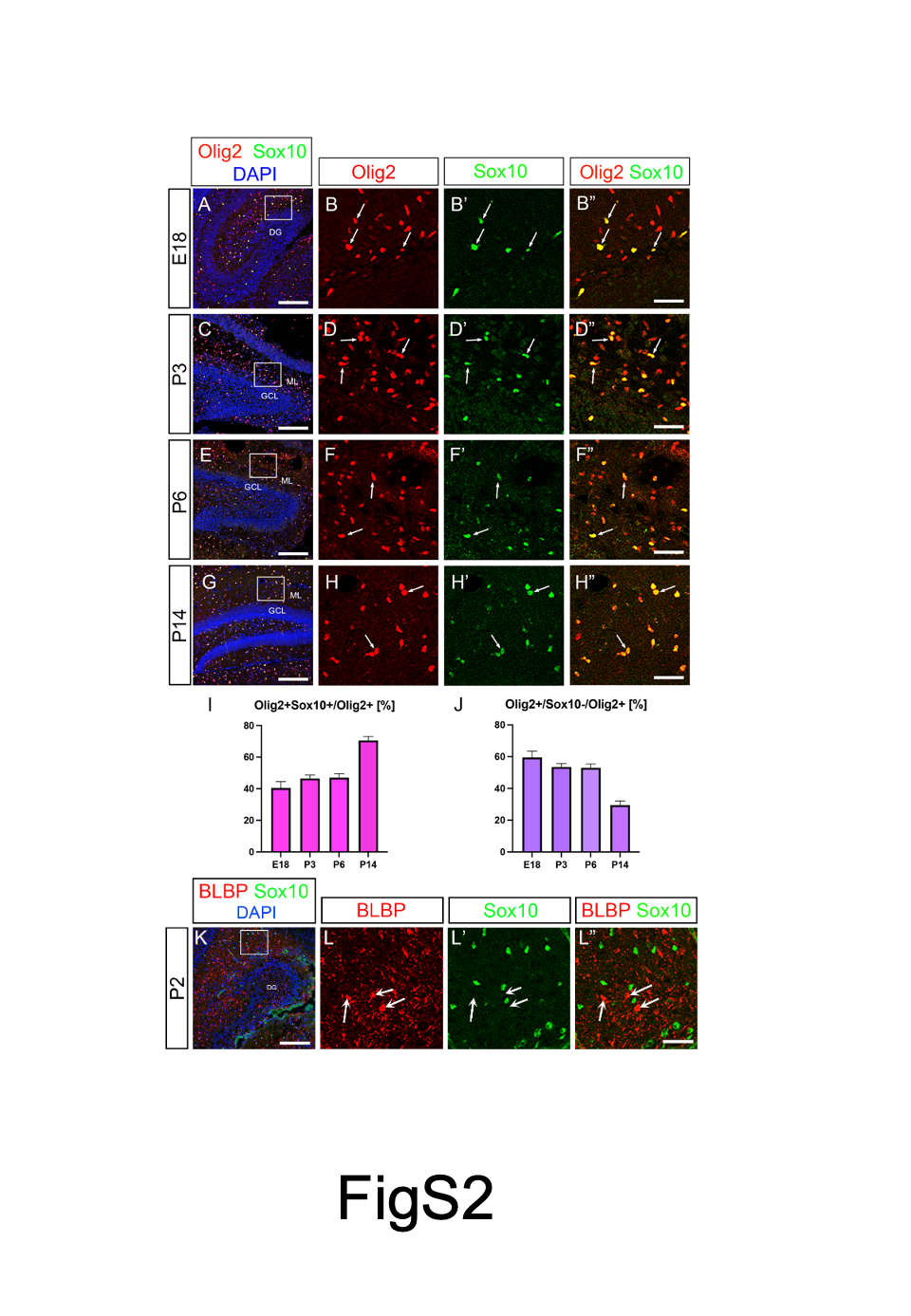

Supplement: Supplementary file 5 [file Image_5.TIFF]

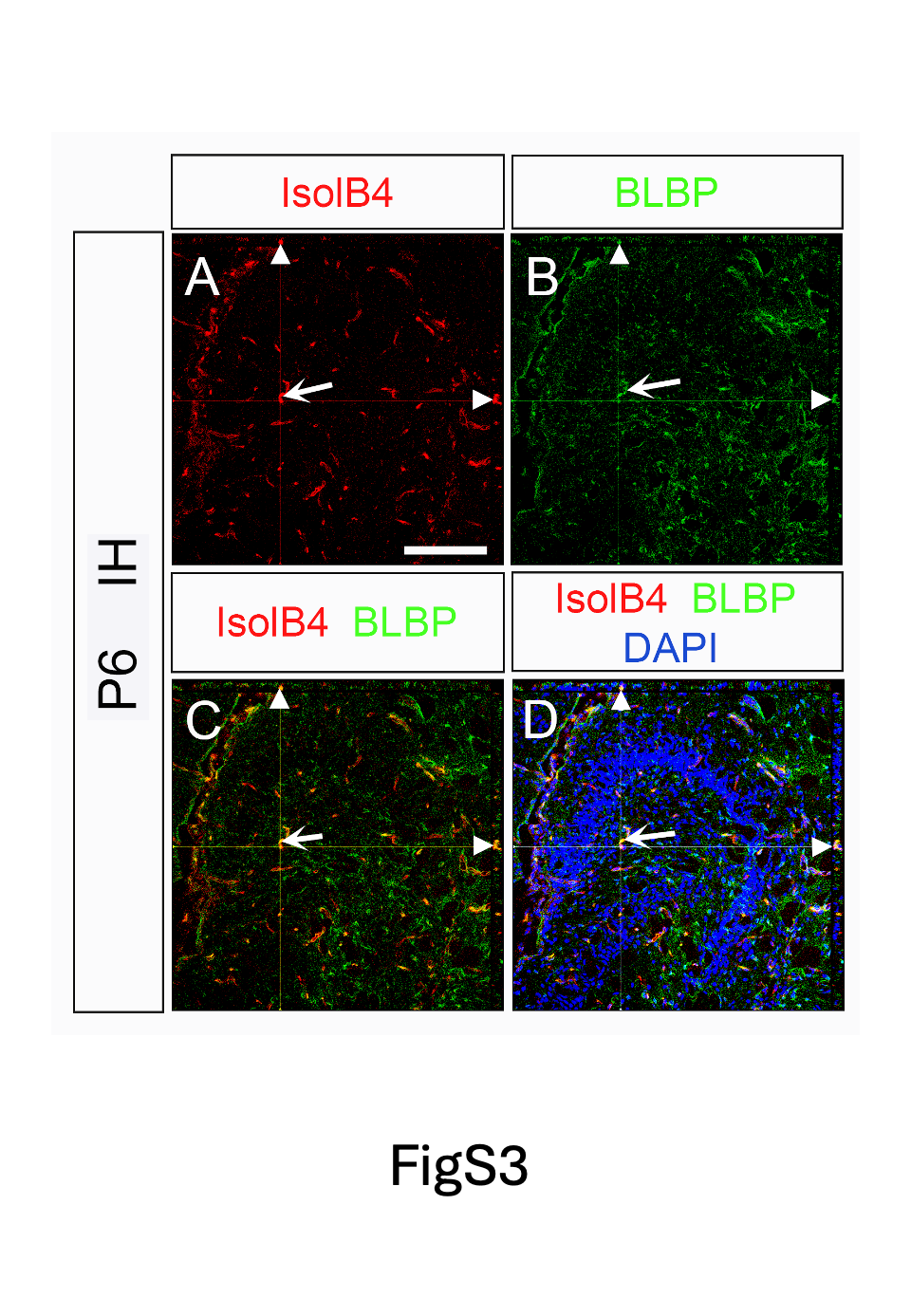

Supplement: Supplementary file 6 [file Image_6.TIFF]
